# Induction of Relaxation by Autonomous Sensory Meridian Response

**DOI:** 10.3389/fnbeh.2021.761621

**Published:** 2021-11-30

**Authors:** Noriko Sakurai, Ken Ohno, Satoshi Kasai, Kazuaki Nagasaka, Hideaki Onishi, Naoki Kodama

**Affiliations:** ^1^Department of Radiological Technology, Niigata University of Health and Welfare, Niigata, Japan; ^2^Department of Physical Therapy, Niigata University of Health and Welfare, Niigata, Japan

**Keywords:** ASMR, auditory perception, relaxation, social behavior, classical music, mPFC

## Abstract

**Background:** Autonomous sensory meridian response (ASMR) is used by young people to induce relaxation and sleep and to reduce stress and anxiety; it comprises somatosensation caused by audiovisual stimuli (triggers) that lead to positive emotions. Auditory stimuli play the most important role among the triggers involved in ASMR and have been reported to be more triggering than visual stimuli. On the other hand, classical music is also known to have a relaxing effect. This is the first study to clarify the difference in brain activation associated with relaxation effects between ASMR and classical music by limiting ASMR to auditory stimulation alone.

**Methods:** Thirty healthy subjects, all over 20 years of age, underwent fMRI while listening to ASMR and classical music. We compared the differences in brain activation associated with classical music and ASMR stimulation. After the experiment, the subjects were administered a questionnaire on somatosensation and moods. After the experiment, the participants were asked whether they experienced ASMR somatosensation or frisson. They were also asked to rate the intensity of two moods during stimulation: “comfortable mood,” and “tingling mood”.

**Result:** The results of the questionnaire showed that none of the participants experienced any ASMR somatosensation or frisson. Further, there was no significant difference in the ratings given to comfort mood, but there was a significant difference in those given to tingling mood. In terms of brain function, classical music and ASMR showed significant activation in common areas, while ASMR showed activation in more areas, with the medial prefrontal cortex being the main area of activation during ASMR.

**Conclusion:** Both classical music and the ASMR auditory stimulus produced a pleasant and relaxed state, and ASMR involved more complex brain functions than classical music, especially the activation of the medial prefrontal cortex. Although ASMR was limited to auditory stimulation, the effects were similar to those of listening to classical music, suggesting that ASMR stimulation can produce a pleasant state of relaxation even if it is limited to the auditory component, without the somatic sensation of tingling. ASMR stimulation is easy to use, and appropriate for wellness purposes and a wide range of people.

## Introduction

Autonomous sensory meridian response (ASMR) videos have gained attention among young people in recent years. ASMR is a type of somatosensation or reaction caused by audiovisual stimuli (Barratt and Davis, 2015; Barratt et al., 2017; Fredborg et al., 2017; McErlean and Banissy, 2017). The triggered response usually extends to the spine, arms, and legs, with a pleasant ASMR somatosensation on the scalp. The purpose of ASMR is to promote sleep, and relaxation, relieve anxiety, and improve work efficiency (Barratt and Davis, 2015; Barratt et al., 2017). The content of ASMR videos varies widely, from gentle whispers in the ear to simulated actions, such as touching the hair or applying makeup, as well as the sounds of chewing, cutting, typing, and nature. Previous research on ASMR analyzed the triggers and identified four prominent categories: whispering, personal attention, vivid sounds, and slow movements; however, these are representative and include many triggers that do not belong to these categories (Barratt and Davis, 2015). Fredborg et al. (2017) then categorized them into five categories (watching, touching, repetitive sounds, simulations, and mouth sounds). The physiological response to ASMR somatosensation is emotionally positive and is accompanied by a sense of calm. The skin conductance response, a measure of autonomic nervous system arousal, is increased, and heart rate is decreased. These seemingly contradictory results reflect the high complexity of ASMR (Poerio et al., 2018).

Functional magnetic resonance imaging (fMRI) has been used to measure brain function. An fMRI study conducted by Lochte et al. (2018) investigated somatosensory brain activity during ASMR stimulation consisting of ASMR videos. ASMR somatosensation involves the activation of secondary somatosensory areas, such as the medial prefrontal cortex, accumbens, insula, inferior frontal gyrus, supplementary motor cortex, and dorsal anterior cingulate cortex (Lochte et al., 2018). Smith et al. also reported on the neural connectivity of the default mode network in subjects who experience the somatosensory component of ASMR and subjects who do not. The DMN of ASMR-sensitive individuals showed decreased connectivity in the precuneus and thalamus, and increased connectivity in the frontal gyrus and temporal gyrus. The study showed differences in brain activation between individuals who experience ASMR and those who do not (Smith et al., 2017). Lee et al. (2020) examined how functional connectivity changes during ASMR video viewing compared to resting state, and assessed its relevance to ASMR-induced emotional states. ASMR-induced changes in emotional state are negatively correlated with functional connectivity for visual information processing.

Personality trait analysis of subjects who have experienced ASMR somatosensation and those who have not has shown that those who experience this sensation are significantly more imaginative, excitable, curious, and open-minded. This is consistent with the personality analysis of those who experience musical frisson (McCrae, 2007; Kovacevichi and Huron, 2018), an emotional response to music and a feeling involving chills and goosebumps (Craig, 2005). The two phenomena, ASMR somatosensation sensation and musical frisson, are similar; however, they differ in that the somatosensation of frisson tends to spread rapidly throughout the body, whereas that of ASMR may last longer than a few minutes (del Campo and Kehle, 2016). In addition, frisson involves an exciting or emotionally stimulating experience; whereas, ASMR somatosensation is more often associated with relaxation and satisfaction (Barratt and Davis, 2015).

Some studies have reported on the wellness benefits of ASMR; ASMR is also already being used in wellness programs and is an efficient way of mind relaxation (Cash et al., 2018). Cash et al. suggest that expectations about the placebo effect of ASMR may lead to somatosensory responses and stress reduction effects. ASMR contributes to home-based stress management and pain management programs due to its ease of use (Cash et al., 2018). The sleep-inducing effects of ASMR have also been proposed as a way to improve sleep quality (Lee et al., 2019; Vardhan et al., 2020). While most studies have considered the somatosensory component of ASMR, this type of sleep induction is limited to auditory stimuli consisting of natural sounds that have no potential to induce ASMR. The combined auditory stimulation of binaural beats for sleeping is measured by electroencephalography, suggesting that this combined stimulation helps the transition to sleep (Lee et al., 2019). Paszkiel et al. (2020) investigated the effects of four different sound stimuli on stress levels as measured using four different methods (EEG, blood pressure, pulse, and questionnaire), and reported that both relaxing music and ASMR induce relaxation at a fast rate and reduce stress levels.

ASMR users do not necessarily experience ASMR somatosensation. However, even in the absence of ASMR somatosensation, mood is improved and chronic pain symptoms are greatly reduced (Barratt and Davis, 2015). Thus, even without ASMR somatosensation, ASMR is being incorporated into wellness programs for the purpose of stress reduction, depressed mood improvement, and pain relief. On the other hand, classical music also is known to have a relaxing effect (Menon and Levitin, 2005), but not all people experience or seek frisson.

This study focuses on the relaxation effects of ASMR and classical music to clarify the differences in brain activation between the two. Auditory stimulation plays the most important role among the triggers that constitute ASMR, and is reported to be more triggering than visual stimulation (Barratt et al., 2017). The present study is the first to clarify the differences in brain activation associated with the relaxation effects listening to classical music vs. the auditory component of ASMR.

## Materials and Methods

### Participants

The subjects of this study were 36 healthy, non-psychiatrically impaired individuals over 20 years of age, who had no experience with ASMR somatosensation, were not enthusiasts, and did not watch ASMR videos for 1 week prior to the experiment. The six participants whose temporal lobe activation could not be confirmed were excluded, as they were probably not listening to the task. Therefore, 30 subjects (18 men and 12 women; mean age, 20.3 years; *SD* = 0.7) were included in the analysis. This study was approved by the Research Ethics Committee of Niigata University of Health and Welfare (Approval No. 18218-190722). Written informed consent was obtained from all participants. Participant interview was performed to ensure the safety of MRI imaging.

### Stimuli Task

**FIGURE 1:**
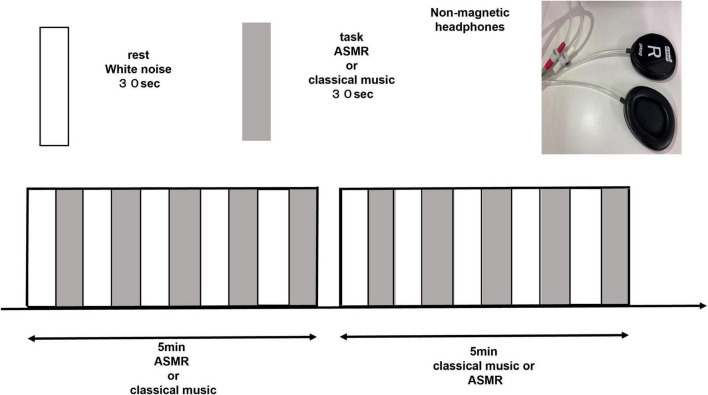
Block design of 5-min with a 30-s rest and a 30-s task repetition (ASMR, classical music). Each subject listened to ASMR stimulation and classical music in random order. ASMR, autonomous sensory meridian response.

We limited the triggers of ASMR to auditory stimuli in order to explore the differences in relaxation effects between ASMR and classical music in terms of brain function. Auditory stimuli are the most important trigger of ASMR and are reported to be more triggering than visual stimuli (Barratt et al., 2017). Lee et al. (2019) used nature sounds as triggers in their sleep-induction experiments, which are limited to auditory stimuli and have no potential for further inducing ASMR. As a sound stimulus task in this study, for ASMR, we selected a sound source with strong auditory effects. We excluded activities of the language cortex, such as whispering, and activities of the visual cortex, such as touching, and prepared 10 patterns of repetitive, crisp, and refreshing sounds. The participants listened to and selected beforehand from among the following sounds: ear scratching, eating a cucumber, typing, pouring soda, stepping in a puddle, grilling a steak, cutting a carrot, wind chimes, flowing water, and rain. Since ASMR is based on personal preference, the subjects listened to all 10 types of ASMR before the experiment and selected the stimulus that they liked the most and that they found most relaxing. Among classical music, Mozart’s music has been reported to be comfortable and to have a great effect on relaxation, resonating with human biological rhythms, balancing the autonomic nervous system, and lowering blood pressure and heart rate (Trappe and Voit, 2016). Thus, we selected Eine kleine Nachtmusik, a piece of music by Mozart that was employed in a previous study (Menon and Levitin, 2005). The resting state task for comparison with the stimulus task was white noise (Cardona et al., 2020). We used white noise because the same auditory stimulus is a necessary condition for the resting task, and because it is a random signal with equal power at any frequency in a given bandwidth without emotional involvement. The block design consisted of a 30-s repetition of the resting task and a 30-s repetition of the stimulus task for a total of 5 min (Figure 1). In fMRI experiments of emotional changes in response to music, music is used as a task in 30-s increments (Pereira et al., 2011). Similarly, in the present study the design was a block of 30-s increments to obtain mood responses without aiming to induce the somatosensory component of ASMR. Subjects were instructed to concentrate on the sound during the 5-min experiment and to keep their eyes open. Cushions were used in the gaps to prevent head movements.

### Apparatus

Imaging was performed on a 3 Tesla MRI system (Canon Vantage Galan) with a 16-channel head coil. The subject laid in the MRI machine and listened to the block design stimuli of ASMR and classical music. The order of stimulus (ASMR or classical music) presentation was determined randomly.

### MRI Acquisition

A separate high-resolution MRI image is required to obtain detailed anatomical information prior to fMRI imaging. For this purpose, a high-resolution magnetization-prepared rapid-gradient-echo sequence of T1-weighted imaging was used, with the following parameters: repetition time, 5.8 ms; echo time, 2.7 ms; inversion time, 900 ms; flip angle, 9°; number of matrices (matrix), 256 × 256; effective field of view, 23 × 23 mm; and slice thickness, 1.2 mm. The echo planar imaging sequence was used to capture the fMRI images. The images were repeatedly obtained, and used to compare the two stimuli. The fMRI imaging conditions were as follows: repetition time, 2,000 ms; echo time, 25 ms; flip angle, 85°; matrix, 64 × 64; effective field of view, 24 × 24 mm; and slice thickness, 3 mm to cover the whole brain.

### Functional Magnetic Resonance Imaging Data Analyses

The fMRI data were preprocessed and analyzed using Statistical Parametric Mapping 12 (Wellcome Trust Center for Neuroimaging) in Matlab (Mathworks Inc.). Slice timing correction was used to correct the time difference, and realignment was then used to correct the displacement caused by motion. In addition, a coregister was used to compare the structural images with the fMRI images. The coregister was corrected for misalignment between structural and functional images and the data was preprocessed by normalizing each participant’s brain to a template of the Montreal Neurological Institute coordinate system of a standard brain. Normalized images were smoothed using a Gaussian kernel of 8 mm. After the preprocessing, we employed a general linear model GLM to confirm brain activity changes associated with ASMR or classical music, using a block design. Contrasts images were created at first level (single-subject) for the following contrast: (1) ASMR = 1, rest = 0, (2) classic = 1, rest = 0, (3) ASMR = 1, classic = −1, respectively. The contrast of (3) was used to identify brain regions with significantly increased activity in the ASMR relative to the classic condition. For group analysis (second level), a one-sample *t*-test was performed using the above three contrasts. The initial threshold for the voxel size was set to uncorrected *p* < 0.001. Clusters were considered significant when falling below *p* = 0.05, cluster-corrected for family wise error. ASMR and classical music were analyzed, and classical music was subtracted from ASMR.

### Questionnaire

After the experiment, the subjects were administered a questionnaire on somatosensation and mood. For somatosensation, the subjects were asked to answer “yes” or “no” to the question of whether they experienced ASMR somatosensation or frisson. They were then asked to indicate the intensity of the two moods for each stimulus: the two moods were “comfortable mood,” and “tingling mood.” A Likert-type scale of 1-5 was used: 1, completely disagree; 2, disagree; 3, undecided; 4, agree; and 5, highly agree. We explained to the subjects that “comfortable mood” refers to a state of relaxation and peace of mind, while “tingling mood” while “tingling” was considered a mood, even in the absence of somatic sensations, even if it does not cause somatic sensations. The chi-square test was performed for the statistical analysis using SPSS (IBM SPSS Statistics Base) 26.0, and the significance level was set at 5%.

## Results

The results of the questionnaire showed that none of the subjects experienced somatosensation during ASMR stimulation; furthermore, none of the subjects experienced musical frisson. The results are shown in Table 1. The results of the ratings of the two moods for each of the ASMR and classical stimuli are shown in Table 2. A 5-point Likert scale questionnaire was used, with 3 points being neutral; the further away the score was from the lower end, the more negative the response was (the mood was not experienced). Conversely, the further away the points were from the higher end, the more positive the response was (the mood was experienced). As for the tingling mood, there was a significant difference between ASMR and classical music stimulation (*p* < 0.001). As for the comfortable mood, there was no significant difference between the ASMR and classical music stimulation (*p* = 0.130). We also found that there is no correlation between comfort and brain activity and between tingling and brain activity.

**TABLE 1:** Frequency of ASMR tingling sensation and musical frisson.

ASMR somatosensation	Classic musical frisson
Yes/No	Yes/No
0/30	0/30

*No subject experienced tingling during ASMR stimulation, and all of them (30) reported not feeling it. No subject experienced musical frisson while listening to classical music, and all of them reported not experiencing it.*

**TABLE 2:** Moods while listening to ASMR and classical music.

Likert scale point		1	2	3	4	5	
Comfortable mood	Classic	3 (10.0%)	2 (6.7%)	5 (16.7%)	14 (46.7%)	6 (20.0%)	*P* = 0.130
	ASMR	2 (6.7%)	9 (30.0%)	6 (20.0%)	11 (36.7%)	2 (6.7%)	
Tingling mood	Classic	6 (20.0%)	14 (46.7%)	2 6.7%)	8 (26.7%)	0 (0.0%)	*P* < 0.001
	ASMR	3 (10.0%)	2 (6.7%)	3 (10.0%)	17 (56.7%)	5 (16.7%)	

*In a comparison of ASMR vs. classical music, there was a significant difference in tingling mood, but there was no significant difference in comfortable mood.*

**FIGURE 2:**
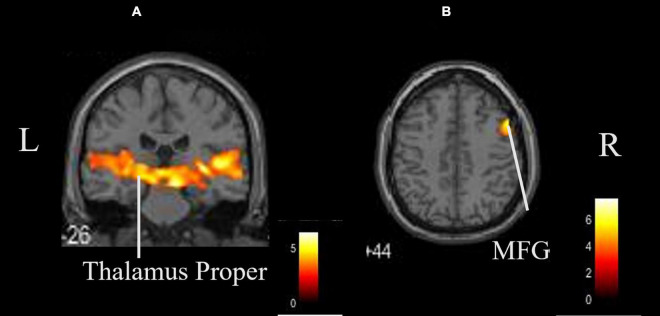
Whole brain activation while listening to classical music compared with the resting state. Significant activation was observed in the right middle frontal gyrus and left thalamus proper. **(A)** Coronal view. **(B)** Axial view. L, left; R, right. MFG, middle frontal gyrus.

**FIGURE 3:**
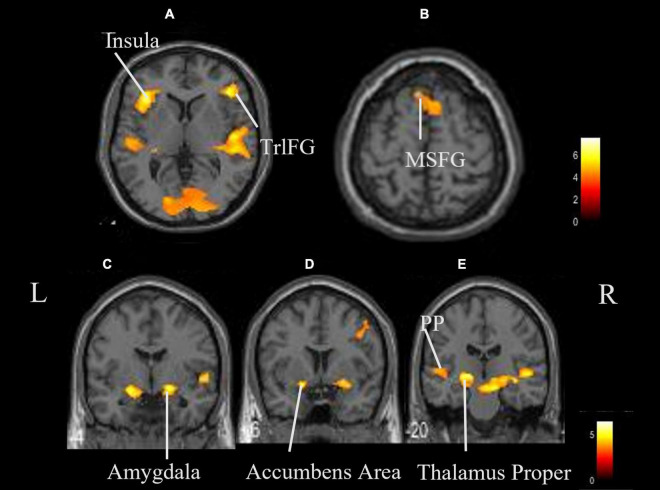
Whole brain activation while listening to ASMR compared with the resting state. Significant activation was observed in the right triangular part of the left thalamus proper, left insula, right triangular part of the inferior frontal gyrus, right cerebellum exterior, left accumbens, right amygdala, left medial superior frontal gyrus, and left planum polare. **(A,B)** Axial view. **(C–E)** Coronal view. L, left; R, right. TrlFG, triangular part of the inferior frontal gyrus; MSFG, medial superior frontal gyrus; PP, planum polare.

**FIGURE 4:**
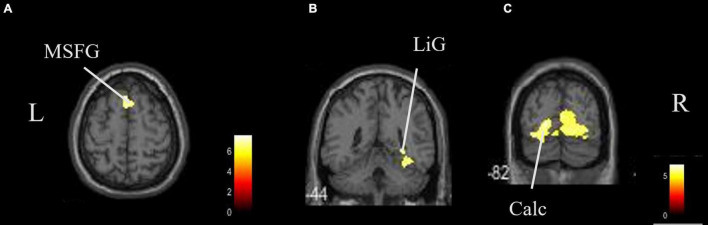
Brain areas that were significantly more activated by ASMR stimulation than by classical music. Significantly more activation was observed in the right triangular part of the left calcarine cortex, right superior frontal gyrus medial segment, and right lingual gyrus in ASMR compared with classical music. **(A)** Axial view. **(B,C)** Coronal view. L, left; R, right. ASMR, autonomous sensory meridian response; LiG, lingual gyrus; MSFG, superior medial gyrus medial segment.

The coordinates of the areas that were significantly activated while listening to classical music are listed in Table 3 and depicted in Figure 2. Significant activation was observed in the right middle frontal gyrus (MFG) and left thalamus proper while listening to classical music. The coordinates of the areas that were significantly activated during ASMR auditory stimulation are listed in Table 4 and depicted in Figure 3. Significant activation was observed in the left thalamus proper, left anterior insula, right triangular part of the inferior frontal gyrus, right cerebellum exterior, left accumbens, right amygdala, left medial superior frontal gyrus (MSFG), and left planum polare. Table 5 shows the coordinates of the areas that were significantly more activated by ASMR stimulation than by classical music. Images of the activated areas are shown in Figure 4. Significant activation was observed in the left calcarine cortex, right superior frontal gyrus medial segment, and right lingual gyrus during ASMR stimulation compared to classical music.

**TABLE 3:** Significantly activated areas and their *Z*-values and coordinates while listening to classical music.

Area	Hemisphere	*K*_E_	*Z*-value	x	y	z (mm)
Thalamus proper	Left	14,823	5.56	−2	−34	−4
Middle frontal gyrus	Right	479	4.79	50	12	46

*Regions of significance after family wise error cluster levels (p < 0.05).*

**TABLE 4:** Significantly activated areas and their *Z*-values and coordinates during autonomous sensory meridian response (ASMR) listening.

Area	Hemisphere	*K*_E_	*Z*-value	x	y	z (mm)
Thalamus proper	Left	2,543	5.01	−30	−26	−2
Insula	Left	648	4.93	−36	24	4
Triangular part of the inferior frontal gyrus	Right	1,142	4.78	44	32	6
Cerebellum exterior	Right	6,219	4.64	26	−64	−24
Accumbens area	Left	293	4.54	−20	2	−12
Amygdala	Right	272	4.52	22	0	−10
Superior frontal gyrus medial segment	Right	457	4.18	6	20	56
Planum polare	Left	262	3.64	−52	−22	4

*Regions of significance after family wise error cluster levels (p < 0.05).*

**TABLE 5:** Significantly activated regions and their *z*-values and coordinates in the difference between autonomous sensory meridian response (ASMR) stimulation and classical music.

Area	Hemisphere	*K*_E_	*Z*-value	x	y	z (mm)
Calcarine cortex	Left	3,596	3.77	−18	−82	0
Superior frontal gyrus medial segment	Right	299	3.72	2	18	58
Lingual gyrus	Right	241	3.68	30	−46	−4

*Regions of significance after family wise error cluster levels (p < 0.05).*

## Discussion

The present study is the first fMRI investigation focusing on the effects of ASMR sound without somatosensory perception on relaxation. We were able to compare the relaxation effects of ASMR with those of classical music by limiting the study to auditory stimuli.

Music is transmitted to the inner ear as air vibrations are captured by the eardrum. It is converted into nerve signals by sensory cells in the inner ear and enters the primary auditory cortex via the brainstem and thalamus. After perceptual processing in the auditory cortex, the information is sent to the association cortex, where it is linked with the parietal and frontal lobes for recognition. The results of this study showed that the thalamus proper and MFG were significantly activated when listening to classical music. The thalamus proper relays auditory sensory nerves to the cerebral cortex and basal ganglia. The same activation was observed in the thalamus while listening to ASMR stimuli. This activation included areas of brain function related to sleep. Sleep begins in the brainstem and enters the thalamus, where auditory sensory information is processed through sensory neural pathways (Tang et al., 2015). Ultimately, it is believed that auditory signals in the thalamus may influence the sleep regulatory system (Lee et al., 2019). The thalamus is involved in emotion and memory, and also acts on the autonomic nervous system. It has been suggested that both classical music and ASMR can induce relaxation and promote sleep.

In addition, more regions were activated during ASMR stimulation. Significant activation was also observed in the anterior insular cortex, nucleus accumbens, amygdala, MSFG, triangular part of the inferior frontal gyrus, and planum polare. The planum polare is located in the temporal lobe, which is the first cortical region of the auditory signal processing system and constitutes the primary auditory cortex. In the basal ganglia and the surrounding limbic system, the amygdala and nucleus accumbens were activated by classical music in addition to the thalamus. The amygdala plays an important role in the control of emotional responses, primarily fear (Ohman, 2005). The nucleus accumbens is involved in reward, satisfaction, and emotion, and is largely responsible for the release of dopamine (Pereira et al., 2011). The insular cortex connects the primary and secondary somatosensory cortices and functions as a multisensory integration site that processes a variety of information (Nagai et al., 2007; Ibañez et al., 2010). In the frontal gyrus, activation was seen in the inferior frontal gyrus and MSFG, indicating that the prefrontal cortex was largely activated; the MSFG is located in the medial prefrontal cortex and has been suggested to contribute to the release of oxytocin (Sabihi et al., 2014).

In this study, we also identified the regions where ASMR stimulation induced a higher activation than classical music. These were the calcarine cortex, superior frontal gyrus medial segment, and lingual gyrus. Activation in the posterior part of the cerebrum is also related to auditory stimulation and is due to spatial hearing (Lewald et al., 2004). We think that ASMR has a more specific impact on the identification of location, direction, and distance of sound sources than classical music.

Particularly noteworthy is the activation of the superior frontal gyrus and the medial prefrontal cortex (mPFC), which is said to be the basis of social cognitive abilities (Feldman, 2012). The mPFC is thought to be activated because ASMR may contain many sound sources that are closely related to daily life and social activity. The mPFC region is also involved in the regulation of neurotransmitters such as dopamine, which projects to the prefrontal cortex through the dopamine pathway and has been shown to enhance stress resistance in response to short-term stress (Tanaka et al., 2012). It also involves the release of oxytocin, which is a stress-reducing, pro-social neuropeptide that is effective in modulating brain activity in depressed individuals (Pincus et al., 2010). These neurohormones are known to induce feelings of comfort, relaxation, and drowsiness (Lochte et al., 2018). ASMR stimulation is thought to be more effective in inducing relaxation and reducing stress than classical music because it activates brain regions associated with these functions.

Lochte et al. (2018) also investigated brain function during moments of relaxation without ASMR somatosensation while watching ASMR videos and found activation in the medial prefrontal cortex. In the present study, ASMR was limited to auditory stimulation, and the results are consistent with those of Lochte et al. (2018). The results of the present study proved using brain functional imaging that the auditory component of ASMR stimulation produced a comfortable and relaxed state, even though the tingling (somatic) sensation was not obtained.

To induce ASMR somatosensation, an individual normally needs a desirable, quiet environment (Barratt and Davis, 2015), which may not always be available. In addition, some persons are more likely than others to experience ASMR somatosensation due to personality traits (Kovacevichi and Huron, 2018). Based on the results of this study, we believe that ASMR stimulation can be used as a tool to easily obtain relaxation just by listening to it, without having to meet demanding requirements. The fact that ASMR is currently being used in wellness for stress reduction, depressive mood improvement, and pain relief further suggests the adequacy of an active use of ASMR. Future studies should examine how listening to ASMR sounds affects brain function in the elderly, and how it can counteract depression in the elderly. The biggest advantages of ASMR include its cost, ease of use, and that it can be completed by an individual at home. This opens up the possibility of proposing the use of ASMR for wellness purposes to an even wider range of persons in the future.

There are several limitations to this study; the results do not provide a quantitative demonstration of the relaxation effect of ASMR. Therefore, we believe that the results of this study can be further strengthened in the future by conducting experiments based on physiological indices and explore correlations. Then, in order to compare ASMR with baseline, we think it is necessary to try to examine a block design with longer rest and task times. Since there comparison group did not used in this study, future studies should compare between groups that are sensitive to ASMR and those that are not. In addition, a subclassification of the ASMR groups might also allow us to deepen our knowledge on this phenomenon. Further investigation of the neurotransmitters suggested in this study, such as dopamine and oxytocin, is needed, and the use of magnetic resonance spectroscopy to obtain information on the type, status, and quantity of neurotransmitters by placing ROIs at the activation sites may further clarify the involvement of specific metabolites.

## Conclusion

ASMR felt as comfortable mood as classical music. In terms of brain function, ASMR stimulation significantly increased activation of mPFC compared to classical music. mPFC is involved in stress reduction and relaxation, suggesting that ASMR can induce relaxation even just by listening to it. Since ASMR stimulation is easy to use, it is expected to expand its use in the future.

## Data Availability Statement

The raw data supporting the conclusions of this article will be made available by the authors, without undue reservation.

## Ethics Statement

The studies involving human participants were reviewed and approved by the Research Ethics Committee of Niigata University of Health and Welfare (Approval Nos. 18218-190722). The patients/participants provided their written informed consent to participate in this study.

## Author Contributions

NS and NK conceived the study and designed the experiments. NS, KO, and NK collected MR data. NS and KN interpreted the data. NS, KO, and KN performed the statistical analysis. SK, HO, and NK helped draft the manuscript. All authors approved the final version of the manuscript.

## Conflict of Interest

The authors declare that the research was conducted in the absence of any commercial or financial relationships that could be construed as a potential conflict of interest.

## Publisher’s Note

All claims expressed in this article are solely those of the authors and do not necessarily represent those of their affiliated organizations, or those of the publisher, the editors and the reviewers. Any product that may be evaluated in this article, or claim that may be made by its manufacturer, is not guaranteed or endorsed by the publisher.

## Abbreviations

ASMR: Autonomous sensory meridian response
MFG: middle frontal gyrus
MSFG: medial superior frontal gyrus.
